# Depression-like phenotype by deletion of α7 nicotinic acetylcholine receptor: Role of BDNF-TrkB in nucleus accumbens

**DOI:** 10.1038/srep36705

**Published:** 2016-11-08

**Authors:** Ji-chun Zhang, Wei Yao, Qian Ren, Chun Yang, Chao Dong, Min Ma, Jin Wu, Kenji Hashimoto

**Affiliations:** 1 Division of Clinical Neuroscience, Chiba University Center for Forensic Mental Health, Chiba 260-8670, Japan

## Abstract

The α7 subtype of nicotinic acetylcholine receptor (nAChR) plays a role in the inflammation which is implicated in depression. This study was undertaken to examine the role of α7 nAChR in depression using α7 nAChR knock-out (KO) mice. Serum levels of tumor necrosis factor-α and interlukin-1β in KO mice were higher than wild-type mice, suggesting an inflammatory process in KO mice. α7 nAChR KO mice showed depression-like phenotype. Furthermore, KO mice showed increased brain-derived neurotrophic factor (BDNF) and its receptor TrkB signaling, as well as increased synaptogenesis and spine density in the nucleus accumbens (NAc), although BDNF-TrkB signaling and synaptogenesis were not altered in the prefrontal cortex and hippocampus. Systemic administration of the TrkB antagonist ANA-12, but not the TrkB agonist 7,8-dihydroxyflavone and the selective serotonin reuptake inhibitor fluoxetine, showed a rapid antidepressant effect in KO mice by normalizing increased synaptogenesis in the NAc. In addition, bilateral infusion of ANA-12 into NAc promoted a rapid antidepressant effect in KO mice by normalizing increased synaptogenesis in the NAc. These findings suggest that increased BDNF-TrkB signaling and synaptogenesis in the NAc by deletion of α7 nAChR plays a key role in depression.

Depression is one of the most common psychiatric disorders in the world. Although the precise mechanism underlying the pathophysiology of depression is unknown, accumulating evidence suggests that inflammation plays a crucial role in depression^1^,^2^,^3^,^4^,^5^,^6^. Meta-analyses show higher blood levels of pro-inflammatory cytokines, such as tumor necrosis factor (TNF)-α and interleukin-6 (IL-6), in drug-free depressed patients, compared with healthy controls^7^,^8^. In addition, a study on postmortem brains revealed elevated gene expression of pro-inflammatory cytokines in the frontal cortex of subjects with a history of major depression^9^. These findings suggest that both peripheral and central inflammation, are associated with depressive symptoms.

Nicotinic acetylcholine receptors (nAChRs) may play a role in the pathophysiology of depression. It is reported that both pharmacological and molecular genetic studies show decreases in hippocampal acetylcholinesterase activity increased anxiety and depression-like behaviors and decreased resilience to repeated stress in a social defeat paradigm^10^, suggesting that abnormalities in the cholinergic system may be critical for the etiology of depression. However, the precise mechanisms underlying the role of nAChRs in depression are not understood^11^,^12^,^13^,^14^. It is known that the α7 subtype of the nAChR regulates the so-called cholinergic ascending anti-inflammatory pathway, in which activation of the vagus nerve diminishes inflammation through decreased peripheral macrophage activity^15^,^16^,^17^,^18^,^19^. Given the role of α7 nAChR in inflammation, it is of great interest to study the role of α7 nAChR in depression associated with inflammation.

Although the pathophysiology of depression remains enigmatic, brain-derived neurotrophic factor (BDNF) and its specific receptor, tropomyosin-receptor-kinase B (TrkB), plays a key role in depression, as well as the therapeutic mechanisms of antidepressants^20^,^21^,^22^,^23^,^24^,^25^,^26^. Preclinical studies suggest that BDNF acts within the ventral tegmental area (VTA) – nucleus accumbens (NAc) pathway, to induce a depression-like phenotype, whereas it produces antidepressant-like effects in the prefrontal cortex (PFC) and hippocampus^6^,^27^,^28^,^29^,^30^,^31^,^32^,^33^,^34^. Currently, there are no reports showing the relationship between α7 nAChR and BDNF–TrkB signaling in depression.

The purpose of this study is to examine whether α7 nAChR plays a role in the pathophysiology of depression. First, we examined whether α7 nAChR knock-out (KO) mice show depression-like phenotype and inflammation. Second, the role of BDNF–TrkB signaling and synaptogenesis in the selected brain regions from α7 nAChR KO mice was examined. Finally, the effects of TrkB agonist 7,8-dihydroxyflavone (7,8-DHF)^35^, TrkB antagonist ANA-12^36^, and selective serotonin reuptake inhibitor (SSRI) fluoxetine in depression-like phenotype of α7 nAChR KO mice were examined.

## Results

### Depression -like phenotype and inflammation of α7 nAChR KO mice

**Figure 1:**
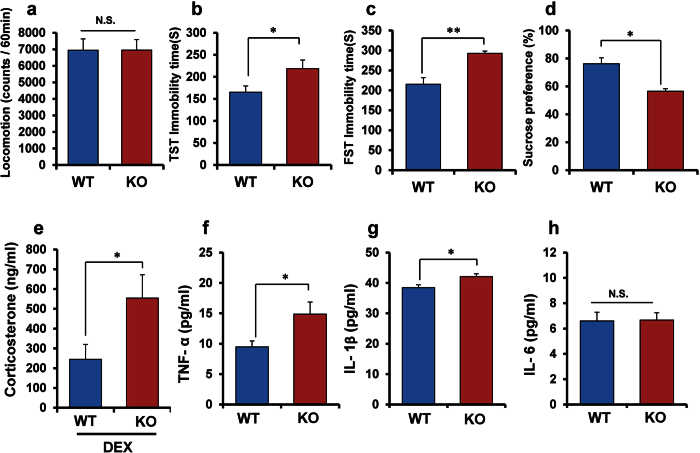
Depression-like phenotypes, and inflammation in α7 nAChR KO mice. (**a**) locomotion, (**b**): tail-suspension test (TST), (**c**): forced swimming test (FST), (**d**): 1% sucrose preference test (SPT). Data represent the mean ± S.E.M. (n = 8 or 9). (**e**): Dexamethasone (DEX) suppression test. Data represent the mean ± S.E.M. (n = 8 or 9). (**f**): Serum levels of TNF-α. Data represent the mean ± S.E.M. (n = 11). (**g**): Serum levels of IL-1β. Data represent the mean ± S.E.M. (n = 7 or 8). (**h**): Serum levels of IL-6. Data represent the mean ± S.E.M. (n = 12 or 13). *P < 0.05, **P < 0.01 compared with the WT group (Student t-test). N.S.: not significant.

Locomotion was no difference between two groups (P = 0.989) (Fig. 1a). In the tail-suspension test (TST) and forced swimming test (FST), the immobility times of α7 nAChR KO mice were significantly (TST: P = 0.042; FST: P = 0.001) higher than those of wild-type (WT) mice (Fig. 1b,c). In the 1% sucrose preference test (SPT), the preference of α7 nAChR KO mice was significantly (P = 0.022) lower than that of WT mice (Fig. 1d), indicative of anhedonia. These data suggest that deletion of α7 nAChR gene causes depression-like phenotypes including anhedonia.

The dexamethasone (DEX) suppression test is an effective way of quantifying dysregulation of the hypothalamic-pituitary-adrenal (HPA) axis, a system involved in depression^33^,^37^,^38^. Serum corticosterone levels in α7 nAChR KO mice were significantly (P = 0.039) higher than those in WT mice six hours after DEX injection (0.1 mg/kg) (Fig. 1e), indicating HPA axis hyperactivity in KO mice. Furthermore, serum levels of TNF-α (P = 0.023) (Fig. 1f) and interlukin-1β (IL-1β) (P = 0.016) (Fig. 1g), but not IL-6 (P = 0.911) (Fig. 1h), in KO mice were significantly higher than those of WT mice, indicating an enhanced inflammatory process in α7 nAChR KO mice.

### Increased BDNF-TrkB signaling and synaptogenesis in the NAc of α7 nAChR KO mice

**Figure 2:**
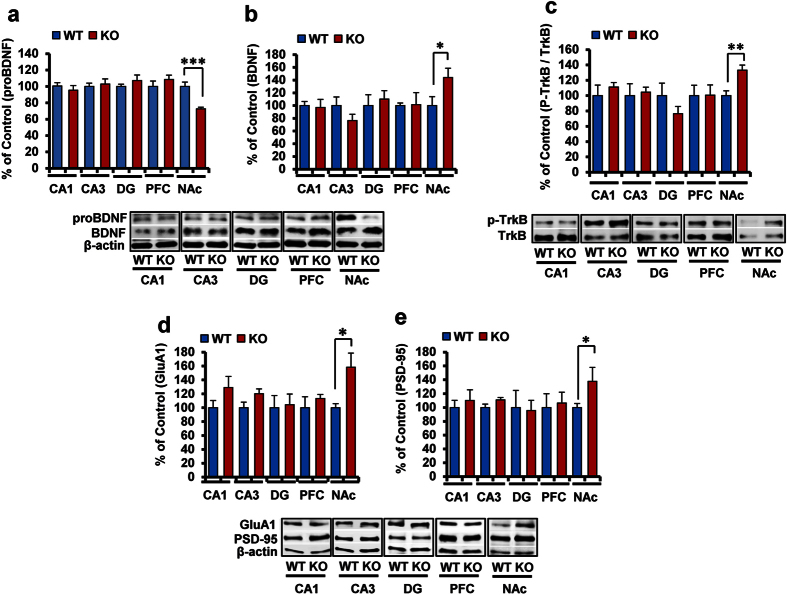
Increased BDNF-TrkB signaling in the NAc of α7 nAChR KO mice. (**a**,**b**): Levels of proBDNF and BDNF in the CA1, CA3, DG, PFC, and NAc. Data represent the mean ± S.E.M. (n = 5 or 6). (**c**): The ratio of phosphor-TrkB to total TrkB. Data represent the mean ± S.E.M. (n = 6). (**d**,**e**): Levels of GluA1 and PSD-95 in the CA1, CA3, DG, PFC, and NAc. Data represent the mean ± S.E.M. (n = 5 or 6). *P < 0.05, **P < 0.01 and ***P < 0.001 compared with the WT group (Student t-test). See the supplemental material 1 for the data of Western blot analyses.

Using Western blot analysis, we examined levels of BDNF and its precursor proBDNF in different brain regions since BDNF-TrkB signaling plays a key role in depression-like phenotype^6^,^27^,^28^,^29^,^30^,^31^,^32^,^33^,^34^. Levels of proBDNF in the NAc of α7 nAChR KO mice were significantly (P < 0.001) lower than those of WT mice, whereas levels of mature BDNF in the NAc of KO mice were significantly (P = 0.048) higher than those of WT mice (Fig. 2a,b). Both proBDNF and BDNF levels in other brain regions, including CA1, CA3, DG and PFC, remained the same (Fig. 2a,b). Furthermore, the ratio of phosphor-TrkB to total TrkB in the NAc of KO mice was also significantly (P = 0.002) higher than that of WT mice although the ratio in other regions was not altered (Fig. 2c).

Next, we measured the markers for synaptogenesis. Levels of the AMPA receptor 1 (GluA1) and postsynaptic density protein 95 (PSD-95) in the NAc of α7 nAChR KO mice were significantly (GluA1: P = 0.027, PSD-95: P = 0.025) higher than those of WT mice, although levels of GluA1 and PSD-95 in other regions were unaltered (Fig. 2d,e).

### Increased dendritic spine density in the NAc of α7 nAChR KO mice

**Figure 3:**
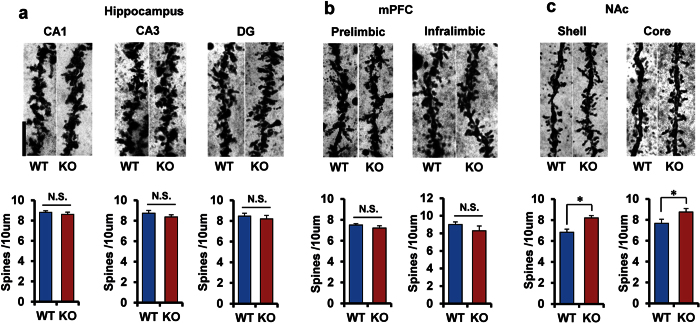
Increased spine density in the NAc of α7 nAChR KO mice. (**a**) CA1, CA3, DG from hippocampus. (**b**) Prelimbic and infralimbic regions of medial prefrontal cortex (mPFC). (**c**) Sell and core regions of NAc. Data represent the mean ± S.E.M. (n = 5). *P < 0.05 compared with the WT group (Student t-test).

Changes in dendritic length and spine density in the brain is thought to contribute to the neurobiology of depression, and antidepressant treatment is mediated, in part, by blocking or reversing these changes^33^,^39^,^40^,^41^. It is, therefore, of interest to examine whether the spine density is altered in the brain regions from α7 nAChR KO mice. Spine density in the CA1, CA3, DG and mPFC (prelimbic and infralimbic areas) was not altered (Fig. 3a,b). In contrast, the spine density in the core and shell of NAc in KO mice was significantly (shell: P = 0.015, core: P = 0.028) higher than that of WT mice (Fig. 3c).

### Antidepressant effect by systemic administration of ANA-12

**Figure 4:**
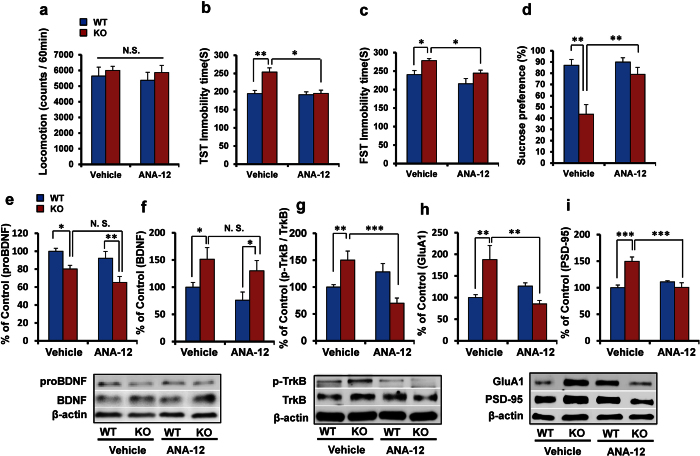
Antidepressant effect of ANA-12, a TrkB antagonist. (**a**): Locomotion, (**b**): TST, (**c**): FST, (**d**): SPT. Data represent the mean ± S.E.M. (n = 8–10). (**e**): proBDNF, (**f**): BDNF, (**g**): p-TrkB/TrkB ratio, (**h**): GluA1, (**i**): PSD-95. Data represent the mean ± S.E.M. (n = 5 or 6). *P < 0.05, **P < 0.01, ***P < 0.001 compared with the WT or KO group (two-way ANOVA). N.S.: not significant. See the supplemental material 2 for the data of Western blot analyses.

Since increased BDNF-TrkB signaling in the NAc was shown in α7 nAChR KO mice, we examined whether the TrkB antagonist ANA-12^31^,^33^,^34^,^36^,^42^,^43^,^44^ could confer antidepressant effects in KO mice. A single dose of ANA-12 (0.5 mg/kg, i.p.) was no effect on locomotion (Fig. 4a). In both the TST and FST, ANA-12 significantly attenuated the increased immobility time of KO mice. Two-way ANOVA analysis revealed significant effects (TST: genotype: F_(1,36)_ = 12.68, P = 0.001, treatment: F_(1,36)_ = 10.46, P = 0.003, interaction: F_(1,36)_ = 7.923, P = 0.008; FST: genotype: F_(1,36)_ = 10.221, P = 0.003, treatment: F_(1,36)_ = 7.723, P = 0.009, interaction: F_(1,36)_ = 0.179, P = 0.675) (Fig. 4b,c). Furthermore, ANA-12 significantly attenuated the reduced sucrose preference of KO mice. Two-way ANOVA analysis revealed significant effects (genotype: F_(1,37)_ = 19.87, P < 0.001, treatment: F_(1,37)_ = 9.876, P = 0.003, interaction: F_(1,37)_ = 7.112, P = 0.012) (Fig. 4d).

Treatment with ANA-12 did not alter protein levels of proBDNF or BDNF in the NAc of all groups (Fig. 4e,f). However, treatment with ANA-12 significantly attenuated the increased ratio of phospho-TrkB to total TrkB in the NAc of α7 nAChR KO mice. Two-way ANOVA analysis revealed significant effects (genotype: F_(1,22)_ = 8.299, P = 0.010, treatment: F_(1,22)_ = 19.28, P < 0.001, interaction: F_(1,22)_ = 9.979, P = 0.005) (Fig. 4g). In addition, ANA-12 significantly attenuated the increased levels of GluA1 and PSD-95 in the NAc of the KO mice. Two-way ANOVA analysis of GluA1 data revealed significant effects (genotype: F_(1,22)_ = 4.832, P = 0.041, treatment: F_(1,22)_ = 7.574, P = 0.013, interaction: F_(1,22)_ = 7.929, P = 0.011). Furthermore, two-way ANOVA analysis of PSD-95 data revealed significant effects (genotype: F_(1,22)_ = 10.26, P = 0.005, treatment: F_(1,22)_ = 9.531, P = 0.006, interaction: F_(1,22)_ = 21.81, P < 0.001) (Fig. 4h,i).

**Figure 5:**
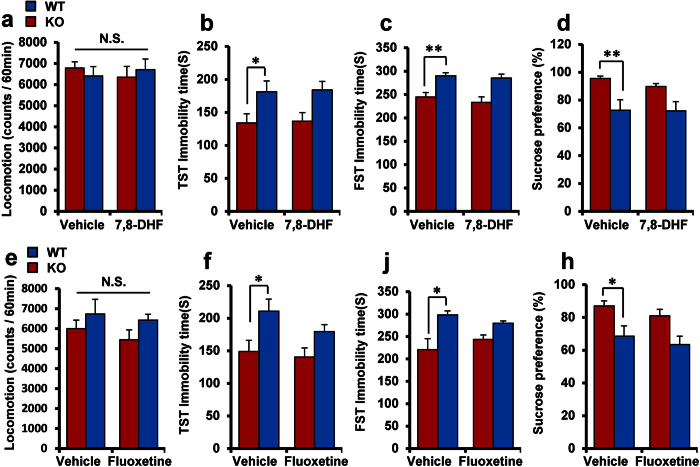
Effects of 7,8-DHF and fluoxetine in α7 nAChR KO mice. (**a**,**e**): locomotion, (**b**,**f**): TST, (**c**,**g**): FST, (**d**,**h**): SPT): The TrkB agonist 7,8-DHF and SSRI fluoxetine did not show antidepressant effects in α7 nAChR KO mice. Data represent the mean ± S.E.M. (n = 12–14). *P < 0.05, **P < 0.01 compared with the vehicle-treated WT group (two-way ANOVA). N.S.: not significant.

In contrast, a single administration with 7,8-dihydroxyflavone (7,8-DHF; 10 mg/kg), a TrkB agonist and selective serotonin reuptake inhibitor (SSRI) fluoxetine (10 mg/kg)^31^,^33^,^34^,^35^,^42^,^43^,^44^,^45^, did not produce antidepressant effects in these mice in TST, FST and 1% SPT (Fig. 5a–h).

### Antidepressant effect by bilateral infusion into NAc of ANA-12

**Figure 6:**
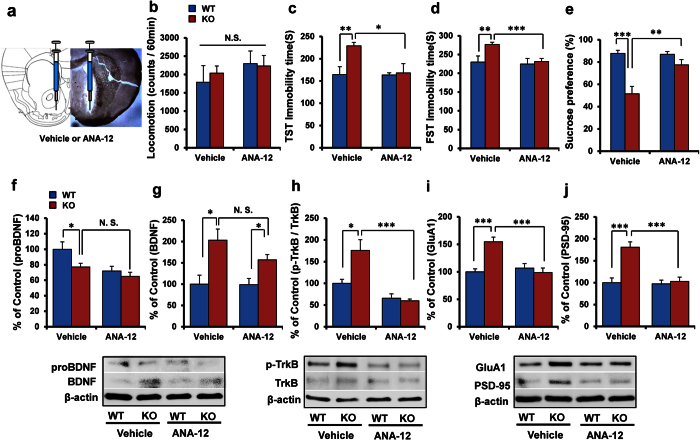
Antidepressant effects of bilateral injection of ANA-12 into NAc shell. (**a**): Scheme of bilateral injection of ANA-12 into NAc shell. (**b**): Locomotion, (**c**): TST, (**d**): FST, (**e**): SPT. Data represent the mean ± S.E.M. (n = 8 or 9). (**f**): proBDNF, (**g**): BDNF, (**h**): p-TrkB/TrkB ratio, (**i**): GluA1, (**j**): PSD-95. Data represent the mean ± S.E.M. (n = 6). *P < 0.05, **P < 0.01, ***P < 0.001 compared with the vehicle-treated KO group (two-way ANOVA). N.S.: not significant. See the supplemental material 2 for the data of Western blot analyses.

In order to confirm the role of increased BDNF-TrkB signaling in the NAc of α7 nAChR KO mice, ANA-12 (0.1 nmol/L, 0.1 μL/min for 5 min) was injected bilaterally into NAc shell of WT or KO mice (Fig. 6a). There was no change in locomotion among four groups (Fig. 6b). Bilateral infusion of ANA-12 significantly attenuated the increased immobility time of KO mice in both TST and FST. Two-way ANOVA analysis revealed significant effects (TST: genotype: F_(1,33)_ = 6.067, P = 0.020, treatment: F_(1,33)_ = 4.852, P = 0.035, interaction: F_(1,33)_ = 4.578, P = 0.041; FST: genotype: F_(1,34)_ = 4.911, P = 0.034, treatment: F_(1,34)_ = 4.390, P = 0.044, interaction: F_(1,34)_ = 3.665, P = 0.065) (Fig. 6c,d). Furthermore, ANA-12 significantly attenuated the reduced sucrose preference of KO mice. Two-way ANOVA analysis revealed significant effects (genotype: F_(1,34)_ = 22.431, P < 0.001, treatment: F_(1,34)_ = 6.809, P = 0.014, interaction: F_(1,34)_ = 7.749, P = 0.009) (Fig. 6e).

Bilateral injection of ANA-12 into NAc-shell of α7 nAChR KO mice did not alter the levels of proBDNF and BDNF in the NAc (Fig. 6f,g). However, bilateral injection of ANA-12 significantly attenuated the increased ratio of phospho-TrkB to total TrkB in the NAc. Two-way ANOVA analysis revealed significant effects (genotype: F_(1,23)_ = 6.032, P = 0.023, treatment: F_(1,23)_ = 27.86, P < 0.001, interaction: F_(1,23)_ = 8.257, P = 0.009) (Fig. 6h). Moreover, bilateral injection of ANA-12 into NAc-shell of α7 nAChR KO mice significantly attenuated the increased levels of GluA1 and PSD-95. Two-way ANOVA analysis of GluA1 data revealed significant effects (genotype: F_(1,23)_ = 9.345, P = 0.006, treatment: F_(1,23)_ = 10.34, P = 0.004, interaction: F_(1,23)_ = 16.89, P = 0.001). Furthermore, two-way ANOVA analysis of PSD-95 data revealed significant effects (genotype: F_(1,23)_ = 16.86, P = 0.001, treatment: F_(1,23)_ = 14.69, P = 0.001, interaction: F_(1,23)_ = 12.90, P = 0.002) (Fig. 6i,j).

## Discussion

The present findings support three important conclusions. First, α7 nAChR KO mice showed depression-like phenotype, including anhedonia. Second, serum levels of TNF-α and IL-1β in α7 nAChR KO mice were higher than those of WT mice, indicative of elevated inflammatory processes in KO mice. Third, we found an increased BDNF-TrkB signaling and synaptogenesis in the NAc (but not PFC and hippocampus) of α7 nAChR KO mice. Interestingly, TrkB antagonist ANA-12, but not TrkB agonist 7,8-DHF and SSRI fluoxetine, showed antidepressant-like effect in KO mice. Interestingly, bilateral infusion of ANA-12 into NAc showed antidepressant effect in KO mice. Taken together, it is likely that the increased BDNF-TrkB signaling in the NAc, but not other brain regions, plays a key role in the depression-like behavior of α7 nAChR KO mice.

Inflammation is known to play a role in depression^1^,^2^,^3^,^4^,^5^,^6^. Peripheral administration of the bacterial endotoxin lipopolysaccharide (LPS) can induce depression-like behavior and alteration in serum pro-inflammatory cytokines in mice^1^,^6^,^46^. Anti-inflammatory drugs and antidepressants, including SSRIs and serotonin and norepinephrine reuptake inhibitors (SNRIs) showed antidepressant effects and alteration pro-inflammatory cytokines in mice after LPS administration^45^,^47^. These findings suggest that peripheral inflammations are associated with depressive symptoms, and it is therefore highly plausible that anti-inflammatory drugs could ameliorate depressive symptoms. In this study, the serum levels of TNF-α and IL-1β, in α7 nAChR KO mice were significantly higher than those of WT mice, indicating that KO mice have inflammation. Thus, it seems that abnormal pro-inflammatory cytokines levels in the serum of α7 nAChR KO mice may play a causative role in the pathophysiology of depression. Given the role of α7 nAChR in the inflammation^17^, treatment with anti-inflammatory compounds might show antidepressant effects in α7 nAChR KO mice, although further detailed studies are needed.

Growing evidence reveals that BDNF in the VTA –NAc pathway is integral to the neurobiology of depression^27^,^28^,^29^,^30^,^31^,^32^,^33^. It was reported that intra-VTA BDNF injections lead to depression-like behavior, while a blockade of BDNF activity in the NAc produced antidepressant effects^29^. Recently, we reported that inflammation and learned helplessness induced a marked increase in BDNF protein within the NAc, resulting in depression-like behavior in rodents^31^,^32^,^33^,^34^. Furthermore, we reported that bilateral injections of ANA-12 into the NAc promoted antidepressant effects in LPS-treated mice^33^ and learned helplessness rats^31^. A study using postmortem brain samples showed increased BDNF levels in the NAc of depression patients, implying a key role for increased BDNF–TrkB signaling in NAc for the development of depression^30^. Thus it seems that abnormal BDNF levels in the VTA–NAc pathways play a causative role in the pathophysiology of depression. In this study, we found the increased BDNF–TrkB signaling in the NAc, but not PFC and hippocampus, of α7 nAChR KO mice, and the TrkB antagonist, ANA-12, but not the TrkB agonist 7,8-DHF, conferred an antidepressant effects by normalizing increased synaptogenesis in the NAc. Furthermore, we found that fluoxetine did not show antidepressant effect in α7nAchR KO mice. It is well known that chronic treatment with SSRIs can increase BDNF levels in the hippocampus and PFC^22^,^23^,^25^,^40^, suggesting that activation of BDNF-TrkB signaling in the PFC and hippocampus, but not in the NAc, might be involved in the antidepressant effects of SSRIs. Taken all together, it is likely that increased BDNF–TrkB signaling in the NAc, due to a deletion of the α7 nAChR gene, may generate the depression-like phenotype seen in α7 nAChR KO mice.

The precise relationship between the α7 nAChR and BDNF is currently unknown. It has been reported that endogenous BDNF is necessary to sustain the distribution patterns of α7 nAChR on the GABAergic hippocampal neurons^48^,^49^, suggesting a close regulation between BDNF and α7 nAChR in the brain. BDNF can enhance neuregulin release at the neuromuscular junction, and neuregulin upregulates functional α7 nAChR on hippocampal neurons^48^. Furthermore, BDNF can increase the number of α7 nAChR clusters on GABAergic hippocampal neurons, suggesting that BDNF can modulate hippocampal output by controlling α7 nAChR levels^49^. In addition, direct injection of nicotine into the posterior ventral tegmental area increased BDNF gene expression in the NAc shell^50^. Moreover, previous studies demonstrated the effect of inflammation on the expression of BDNF in the brain^33^. We reported that administration of LPS significantly increased BDNF protein expression in the NAc^33^. Given the role of α7 nAChR in inflammation, it is likely that inflammation might increase BDNF protein expression in the NAc of α7 nAChR KO mice. Further detailed study on the mechanisms underlying increased BDNF-TrkB signaling in the NAc by deletion of α7 nAChR gene is needed.

Tracking dendritic morphology, revealed increased spine density in the NAc of α7 nAChR KO mice. Recently, we reported increased spine density and BDNF in the NAc of the inflammation-induced model of depression in mice^33^, learned helplessness rats^31^,^32^, social defeat stress in mice^51^, and methamphetamine withdrawal^42^. Given that synaptogenesis is a key function in the mechanism of antidepressants^39^,^40^,^41^, the therapeutic effect of ANA-12 on depression-like behavior and the increased spine density seen in NAc of α7 nAChR KO mice are of great interest. It is also therefore likely that α7 nAChR KO mice may be an animal model for depression to screen new TrkB antagonists.

In conclusion, these findings suggest that increased BDNF–TrkB signaling in the NAc plays a key role in depression phenotype of α7 nAChR KO mice. Therefore, TrkB antagonists could represent novel therapeutic drugs for depressed patients with increased BDNF–TrkB signaling in the NAc.

## Methods and Materials

### Animals

Mice deficient for the α7 nAChR (C57BL/6 background) were purchased from The Jackson Laboratory (Bar Harbor, ME). Male wild-type (WT) and knock-out (KO) mice aged 2–3 months were used for experiment. The mice were housed in clear polycarbonate cages (22.5 × 33.8 × 14.0 cm) in groups of 5 or 6 per cage under a controlled 12/12-h light–dark cycle (lights on from 7:00 a.m. to 7:00 p.m.), with a room temperature of 23 ± 1 °C and humidity of 55 ± 5%. All experiments were carried out in accordance with the Guide for Animal Experimentation of Chiba University. The protocol was approved by the Chiba University Institutional Animal Care and Use Committee.

### Drug administration

On the day of injection, fresh solutions were prepared by dissolving compounds in sterile endotoxin-free isotonic saline. ANA-12, N2-(2-{[(2-oxoazepan-3-yl) amino]carbonyl}phenyl)benzo[b] thiophene-2-carboxamide (0.5 mg/kg, Catalog number: BTB06525SC, Maybridge), was dissolved in 1% dimethylsulfoxide (DMSO) in physiological saline. 7,8-Dihydroxyflavone (7,8-DHF; 10 mg/kg, Catalog number: D1916, Tokyo Chemical Industry, Tokyo, Japan) was prepared in a vehicle of 17% DMSO in phosphate-buffered saline. Fluoxetine (10 mg/kg, Wako Chemical Co., Ltd, Tokyo, Japan) was prepared in physiological saline. 7,8-DHF and ANA-12 was administered intraperitoneally (i.p.). The doses of 7,8-DHF, ANA-12 and fluoxetine were selected as previously reported^33^,^34^,^42^,^43^,^44^,^45^.

### Behavioral tests

Behavioral tests were performed as reported previously^33^,^34^. Locomotion: the mice were placed in experimental cages (length × width × height: 560 × 560 × 330 mm). Locomotor activity of mice was counted by the SCANETMV-40 (MELQUEST Co., Ltd., Toyama, Japan), and cumulative exercise was recorded for 60 minutes. Cages were cleaned between testing session. Tail suspension test (TST): The mice were taken from their home cage and a small piece of adhesive tape was placed approximately 2 cm from the tip of their tail. A single hole was punched in the tape and mice were hung individually, on a hook. The immobility time of each mouse was recorded for 10 minutes. Mice were considered immobile only when they hung passively and completely motionless. Forced swimming test (FST): The mice were placed individually in a cylinder (diameter: 23 cm; height: 31 cm) containing 15 cm of water, maintained at 23 ± 1 °C. Animals were tested in an automated forced-swim apparatus using SCANETMV-40 (MELQUEST Co., Ltd., Toyama, Japan). Immobility time was calculated from activity time as (total) – (active) time, using the apparatus analysis software. Cumulative immobility time was scored for 6 minutes during the test. Sucrose preference test (SPT): Mice were habituated to a 1% sucrose solution for 48 h before the test day. Mice were deprived of water and food for 4 h, followed by a preference test spanning 1 h with water and 1% sucrose, delivered from identical bottles. The bottles containing water and sucrose were weighed before and at the end of this period and the sucrose preference was determined.

### Dexamethasone (DEX) suppression test

Dexamethasone (DEX: Wako Pure Chemical Co., 0.1 mg/kg) was injected i.p. into mice, as reported previously^33^,^37^. Six hours after injection of DEX, mice were anesthetized with pentobarbital, and blood was collected from heart. Blood was centrifuged at 2000 g for 20 minutes to generate serum samples. The serum levels of corticosterone were measured using an Assay MAX corticosterone ELISA kit (St. Charles, MO).

### Measurement of pro-inflammatory cytokines

The WT mice and KO mice were anesthetized with pentobarbital, and blood was collected from heart. Blood was centrifuged at 2000 g for 20 minutes to generate serum samples. The serum samples were diluted 10-fold with ELISA diluent solution (eBioscience, San Diego, CA, USA). The serum levels of TNF-α, IL-1β or IL-6 concentrations were measured using a Ready-SET-Go ELISA kit (eBioscience) according to the manufacturer’s instructions.

### Western blot analysis of proBDNF, BDNF, p-TrkB, TrkB, PSD-95 and GluA1

The WT and KO mice brain samples of CA1, CA3 and dentate gyrus (DG) of the hippocampus, prefrontal cortex (PFC) and nucleus accumbens (NAc) were collected as previously reported^32^,^33^,^34^. For the ANA-12 treated mice, the brain samples (NAc) were collected after SPT. Tissue samples were homogenized in Laemmli lysis buffer. Aliquots (10 μg for mouse tissue) of protein were measured using the DC protein assay kit (Bio-Rad, Hercules, CA), and incubated for 5 min at 95 °C, with an equal volume of 125 mM Tris/HCl, pH 6.8, 20% glycerol, 0.1% bromophenol blue, 10% β-mercaptoethanol, 4% sodium dodecyl sulfate, and subjected to sodium dodecyl sulfate polyacrylamide gel electrophoresis, using 10% mini-gels (Mini-PROTEAN® TGX™ Precast Gel; Bio-Rad, CA). Proteins were transferred onto polyvinylidenedifluoride (PVDF) membranes using a Trans Blot Mini Cell (Bio-Rad). For immunodetection, the blots were blocked with 2% BSA in TBST (TBS + 0.1% Tween-20) for 1 h at room temperature (RT), and kept with primary antibodies overnight at 4 °C. The following primary antibody was used: BDNF (1: 200, Santa Cruz Biotechnology, Inc., CA), phosphor-TrkB (Tyr 706) (1:200, Santa Cruz Biotechnology, Inc., CA), TrkB (80E3) (1:1000, Cell Signaling Technology, MA), postsynaptic density protein 95 (PSD-95) (1 μg/ml Invitrogen, Carlsbad, CA), and glutamate receptor 1 (GluA1) (1 μg/ml Abcam, Cambridge, MA). The BDNF antibody recognized both proBDNF and mature BDNF. The next day, blot were washed three times in TBST and incubated with horseradish peroxidase conjugated anti-rabbit antibody (1:1000 for TrkB and 1:5000 for BDNF, PSD-95 and GluA1) and goat anti-rabbit antibody (1:2000 for p-TrkB) 1 hour, at RT. After final three washes with TBST, bands were detected using enhanced chemiluminescence (ECL) plus the Western Blotting Detection system (GE Healthcare Bioscience). The blots then were incubated in the stripping buffer (2% SDS, 100 mM β-mercaptoethanol, 62.5 mM Tris/HCL PH 6.8) for 30 min at 60 °C followed by three time washed with TBST. The stripped blots were kept blocking solution for 1 hour and incubated with the primary antibody directed against TrkB protein, GluA1 protein and β-Actin. Images were captured with a Fuji LAS3000-mini imaging system (Fujifilm, Tokyo, Japan), and immunoreactive bands were quantified.

### Golgi staining

Golgi staining was performed using the FD Rapid GolgiStain^TM^ Kit (FD Neuro Technologies, Inc., Columbia, MD), following the manufacturer’s instructions. Animals were deeply anesthetized with sodium pentobarbital, and brains were removed from the skull and rinsed in double distilled water. Brains were immersed in the impregnation solution, made by mixing equal volumes of Solution A and B, overnight and then stored in fresh solution, for 2 weeks in the dark. Brains were transferred into Solution C overnight and then stored in fresh solution at 4 °C for 1 week, in the dark. Coronal brain sections (100 μm thickness) were cut on a cryostat (3050S, Leica Microsystems AG, Wetzlar, Germany), with the chamber temperature set at −20 °C. Each section was mounted in Solution C, on saline-coated microscope slides. After absorption of excess solution, sections were dried naturally, at room temperature. Dried sections were processed following the manufacturer’s instructions. Briefly, images of dendrites within CA1, CA3, and DG of the hippocampus, PFC and NAc were captured using a 100x objective with a Keyence BZ-9000 Generation II microscope (Osaka, Japan). Spines were counted along CA1, CA3, DG, PFC and NAc dendrites starting from their point of origin from the primary denarite, as previously reported^32^,^33^. For spine density measurements, all clearly evaluable areas containing 50–100 μm of secondary dendrites from each imaged neuron were used. To determine relative spine density, spines on multiple dendritic branches from a single neuron were counted to obtain an average spine number per 10 μm. For spine number measurements, only spines that emerged perpendicular to the dendritic shaft were counted. Three neurons per section, three sections per animal and five animals were analyzed. The average value for each region, in each individual was obtained. These individual averages were then combined to yield a grand average for each region.

### Systemic administration of ANA-12, 7,8-DHF and fluoxetine

ANA-12 (0.5 mg/kg), 7,8-DHF (10 mg/kg), fluoxetine (10 mg/kg) or vehicle (10 ml/kg) was administered i.p. into α7 nAChR KO mice or WT mice. Behavioral evaluation was performed at 1 hour (locomotion), 4 hours (TST) and 6 hours (FST). The SPT was performed at 36 hours after a single administration.

### Bilateral injection of ANA-12 into NAc

Mice were anesthetized with pentobarbital (5 mg/ml, 0.15 ml/mouse), and placed in a stereotaxic frame. Microinjection needles were placed bilaterally into the NAc shell (+1.7 AP, ± 0.75 ML, −3.6 DV)^33^. Twenty-four hours after surgery, ANA-12 (0.1 nmol/L, 0.1 μL/min for 5 min) or vehicle was injected bilaterally. Behavioral evaluation was performed at 1 hour (locomotion), 4 hours (TST) and 6 hours (FST), the SPT was performed at 36 hours after the final infusion.

### Statistical analysis

The data are show as the mean ± standard error of the mean (SEM). Analysis was performed using PASW Statistics 20 (formerly SPSS Statistics; SPSS). Comparisons between groups were performed using the Student’s t-test and two-way ANOVA, when appropriate, *post hoc* comparisons were performed using the unpaired t-test. The P-values of less than 0.05 were considered statistically significant.

## Additional Information

**How to cite this article:** Zhang, J.-C. *et al*. Depression-like phenotype by deletion of α7 nicotinic acetylcholine receptor: Role of BDNF-TrkB in nucleus accumbens. *Sci. Rep.* **6**, 36705; doi: 10.1038/srep36705 (2016).

## Supplementary Material

Supplementary Information
