# Supplementary material for: Depression-like phenotype by deletion of α7 nicotinic acetylcholine receptor: Role of BDNF-TrkB in nucleus accumbens
Source: Sci Rep. 2016 Nov 8;6:36705. doi: 10.1038/srep36705 (PMC5099687; doi:10.1038/srep36705)

Supplemental figure legends and figures

Depression-like phenotype by deletion of α7 nicotinic acetylcholine receptor: Role of BDNF-TrkB in nucleus accumbens

Ji-chun Zhang, Wei Yao, Qian Ren, Chun Yang, Chao Dong, Min Ma,
Jin Wu and Kenji Hashimoto

Division of Clinical Neuroscience, Chiba University Center for Forensic Mental Health, Chiba 260-8670, Japan

Correspondence: Kenji Hashimoto, Division of Clinical Neuroscience, Chiba University Center for Forensic Mental Health, 1-8-1 Inohana, Chiba 260-8670, JAPAN.

Supplemental figure legends

Supplemental figure 1: The western blot of proteins in the selected brain regions from α7 nAChR WT and KO mice.
(a): The blots for proBDNF, BDNF and actin.
(b): The blots for p-TrkB and TrkB.
(c): The blots for GluA1, PSD-95 and actin.
The red line marked blots were selected in the figure 2.

Supplemental figure 2: The western blot of proteins in the NAc from α7 nAChR WT and KO mice after ANA-12 injection.
(a): The blots for proBDNF, BDNF and actin in the NAc after i.p. injection of ANA-12.
(b): The blots for p-TrkB, TrkB and actin in the NAc after i.p. injection of ANA-12.
(c): The blots for GluA1, PSD-95 and actin in the NAc after i.p. injection of ANA-12.
(d): The blots for proBDNF, BDNF, and actin in the NAc after bilateral injection of ANA-12 into the NAc.
(e): The blots for p-TrkB, TrkB and actin in the NAc after bilateral injection of ANA-12 into the NAc.
(f): The blots for GluA1, PSD-95 and actin in the NAc after bilateral injection of ANA-12 into the NAc.
The red line marked blots were selected in the figure 4 and figure 6.

Supplemental figure 1

a. For figure 2a and b

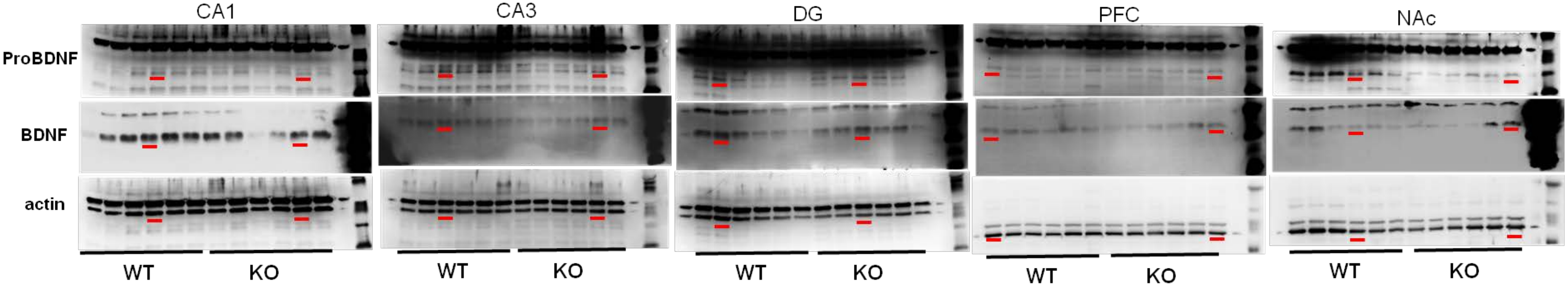

b. For figure 2c

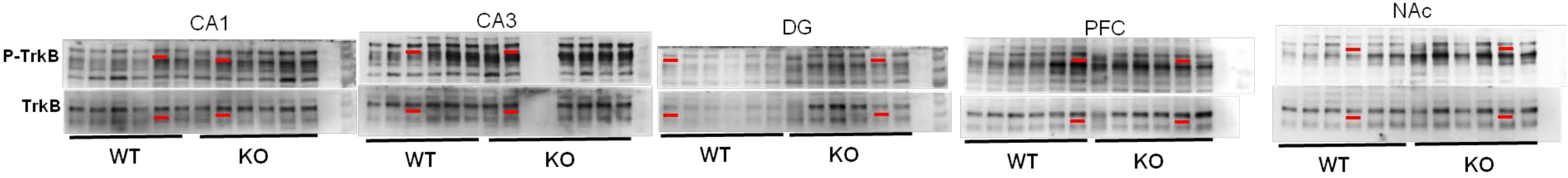

c. For figure 2d and e

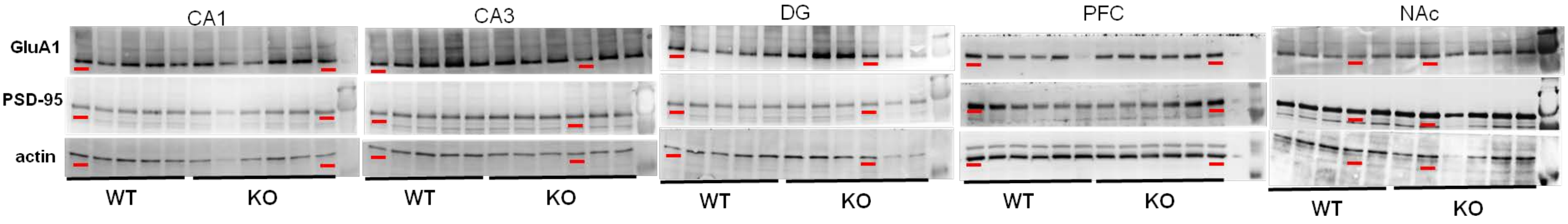

Supplemental figure 2

a. For figure 4e

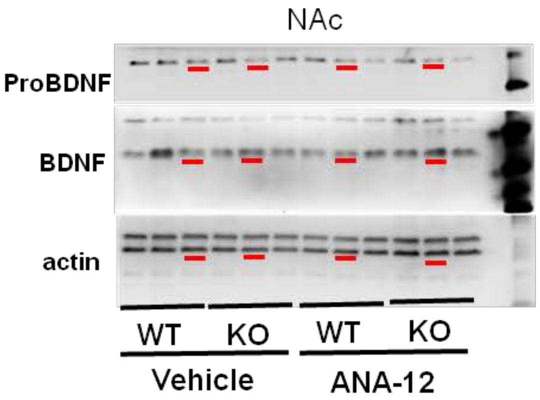

b. For figure 4g

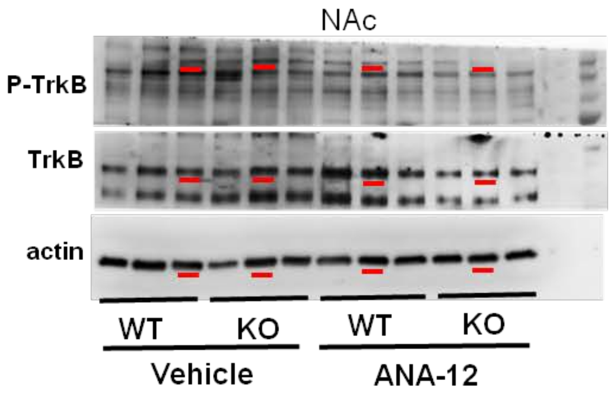

c. For figure 4h and i

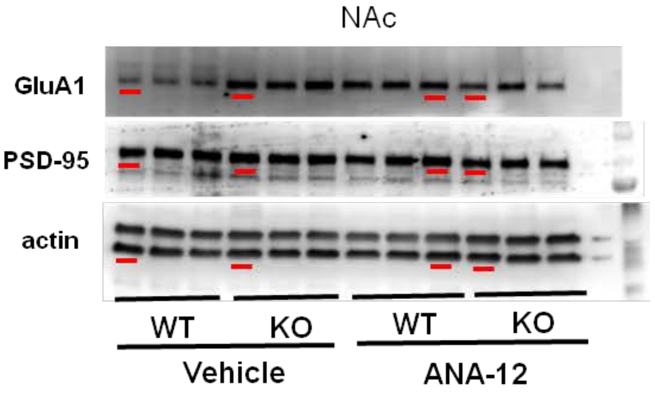

d. For figure 6f and g

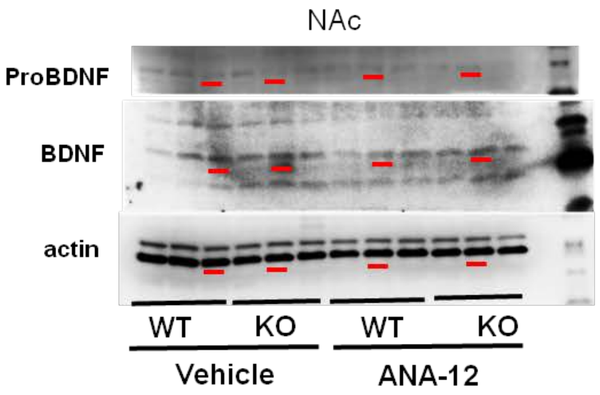

e. For figure 6h

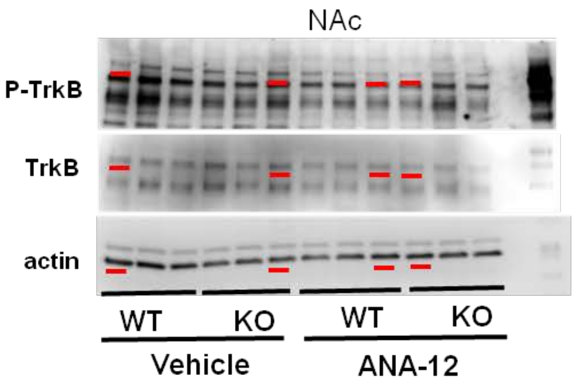

f. For figure 6i and j

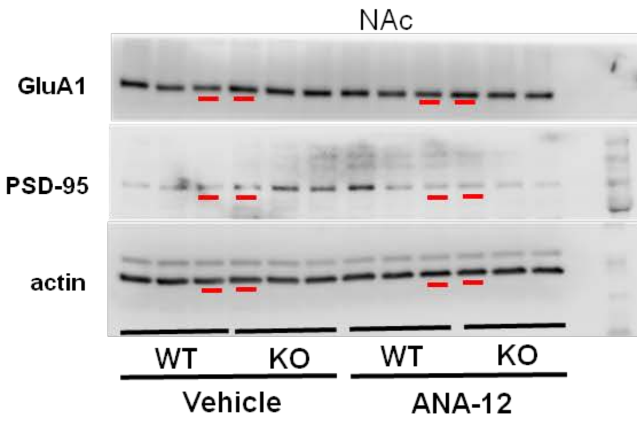

Supplement: Supplementary Information [file srep36705-s1.pdf]
